# Deep Learning Algorithms for Bladder Cancer Segmentation on Multi-Parametric MRI

**DOI:** 10.3390/cancers16132348

**Published:** 2024-06-26

**Authors:** Kazim Z. Gumus, Julien Nicolas, Dheeraj R. Gopireddy, Jose Dolz, Seyed Behzad Jazayeri, Mark Bandyk

**Affiliations:** 1 Department of Radiology, College of Medicine-Jacksonville, University of Florida, Jacksonville, FL 32209, USA; 2 Laboratory for Imagery, Vision and Artificial Intelligence, ETS Montreal, Montreal, QC H3C 1K3, Canada; 3 Department of Urology, College of Medicine-Jacksonville, University of Florida, Jacksonville, FL 32209, USAmark.bandyk@jax.ufl.edu (M.B.)

**Keywords:** bladder cancer, segmentation, MRI, deep learning, loss function, Unet, MAnet, PSPnet, cross-entropy, focal loss, expected calibration error

## Abstract

**Simple Summary:**

Bladder cancer segmentation on MRI images is critical to determine if the cancer spread to the nearby muscles. In this study, we aimed to assess the performance of three deep learning models in outlining bladder tumors from MRI images. Using the MRI data of 53 patients, we trained Unet, MAnet, and PSPnet models to segment tumors using different loss functions and evaluated their performances. The results showed MAnet and PSPnet models performed better overall in segmenting bladder tumors, especially when they used a hybrid loss function (CE+DSC). Our findings could improve the way bladder cancer is segmented on MRI images, potentially leading to a better choice of deep learning algorithms and loss functions for future research.

**Abstract:**

Background: Bladder cancer (BC) segmentation on MRI images is the first step to determining the presence of muscular invasion. This study aimed to assess the tumor segmentation performance of three deep learning (DL) models on multi-parametric MRI (mp-MRI) images. Methods: We studied 53 patients with bladder cancer. Bladder tumors were segmented on each slice of T2-weighted (T2WI), diffusion-weighted imaging/apparent diffusion coefficient (DWI/ADC), and T1-weighted contrast-enhanced (T1WI) images acquired at a 3Tesla MRI scanner. We trained Unet, MAnet, and PSPnet using three loss functions: cross-entropy (CE), dice similarity coefficient loss (DSC), and focal loss (FL). We evaluated the model performances using DSC, Hausdorff distance (HD), and expected calibration error (ECE). Results: The MAnet algorithm with the CE+DSC loss function gave the highest DSC values on the ADC, T2WI, and T1WI images. PSPnet with CE+DSC obtained the smallest HDs on the ADC, T2WI, and T1WI images. The segmentation accuracy overall was better on the ADC and T1WI than on the T2WI. The ECEs were the smallest for PSPnet with FL on the ADC images, while they were the smallest for MAnet with CE+DSC on the T2WI and T1WI. Conclusions: Compared to Unet, MAnet and PSPnet with a hybrid CE+DSC loss function displayed better performances in BC segmentation depending on the choice of the evaluation metric.

## 1. Introduction

Artificial intelligence (AI) applications are being adapted for medical imaging in radiology. AI models, more specifically deep learning (DL) convolutional neural networks (CNN), have illustrated remarkable success in the interpretation of medical images with computer-aided detection and localization of imaging abnormalities. However, clinicians not understanding the novel methods may fail to incorporate the technology into daily practice and accept computer-aided interpretation. Using multi-parametric magnetic resonance images (mp-MRI) of bladder cancer (BC), this work illustrates how incorporating DL models benefits the diagnosis and evaluation of BC. 

BC is the 10th predominant cancer in the world according to the World Cancer Research Fund International [1]. Risk factors are smoking, parasitic infections (schistosomiasis), and toxic chemicals such as aromatic amines (occupational exposure) and arsenic (drinking water). BC is the fourth most common cancer among elderly men in the US [2]. The American Cancer Society estimates that there will be 83,190 new cases of bladder cancer and 16,840 deaths from bladder cancer in 2024 [3]. Early bladder cancer diagnosis, accurate staging, and surgical treatment reduce the morbidity and mortality of bladder cancer. With the advances in surgical techniques, chemotherapy, immunotherapy, and diagnostic imaging options, bladder cancer mortality has had a declining trend in the last 5 years [4]. 

**Figure 1:**
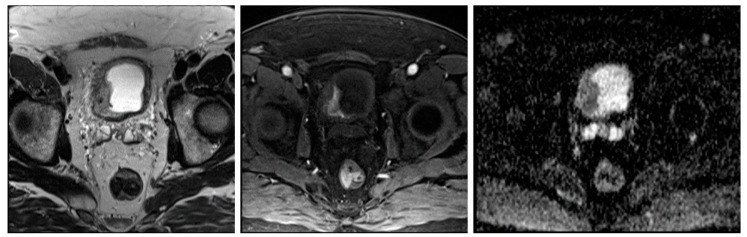
A bladder cancer case is depicted. An axial T2-weighted MRI shows an abnormal signal in the bladder (**left**). Post-contrast image reveals focal enhancement in the arterial phase (**middle**), while the ADC diffusion map indicates a low signal consistent with a high-grade tumor (**right**).

The determination of muscle invasion in BC guides proper risk stratification and therapy [5,6,7,8]. Currently, the gold standard of bladder cancer staging is transurethral resection of the bladder tumor (TURBT). TURBT enables the pathologic diagnosis and staging of muscle-invasive bladder cancer (MIBC). However, up to 30% of TURBT specimens are inaccurate, and the staging of bladder cancer changes in a repeat TURBT. Accurate, non-invasive imaging of bladder cancer could help eliminate the shortcomings of this staging surgical procedure. From its initial description in 1962 [9], this surgical procedure has evolved little with potential complications and limitations. Recent clinical outcome data indicate that a high-quality TURBT requires experience, clinical judgment, precise tumor resection technique, or sometimes repeating the TURBT. A TURBT has up to 6.7% complications, including bladder perforation and uncontrolled bleeding risk [10]. While repeat resection detects residual cancer in 26 to 83% of patients [11], occult locally advanced (extravesical) cancer cannot be detected by repeat TURBT. In fact, cross-sectional imaging is recommended in the follow-up of patients who are managed only with TURBT to rule out locally growing extravesical disease processes.

mp-MRI is an evolving tool for bladder cancer staging [12]. mp-MRI imaging allows for high soft tissue contrast resolution and multiplanar imaging, enabling radiologists to predict the depth of tumor invasion (Figure 1) [13,14,15]. Detecting the presence or absence of MIBC is the critical step in risk stratification and therapy of bladder cancer [6]. Utilizing DL potentially would improve the accuracy and automate bladder cancer mp-MRI segmentation [12]. 

However, current mp-MRI requires improvements in the accuracy, efficiency, and consistency of BC staging. It lacks reliable discrimination of muscle invasion [15,16,17,18]. Slice-by-slice MRI evaluations are tedious, and the effectiveness depends upon the experience of the radiologist. Accurate interpretation of mp-MRI images can be complicated by motion artifacts, bladder wall inflammation, and degrees of bladder distension. 

Similar to PI-RADS for the prostate and BI-RADS for the breast, vesicle imaging reporting and data systems (VI-RADS) have been implemented for bladder imaging. VI-RADS standardizes MRI interpretation to detect MIBC. When using VI-RADS scores for mp-MRI, the first parameter evaluated is diffusion-weighted/apparent diffusion coefficient images (DWI/ADC), followed by contrast-enhanced T1-weighted (T1WI) and T2-weighted images (T2WI). DWI/ADC is the most important sequence for BC staging [15]. However, DWI images are susceptible to artifacts; hence, T1WI and T2WI are relied upon to determine MIBC. Due to inflammation in the bladder wall or fibrosis on T2WI, there can also be false positive MIBC [19]. The T1WI sequence may not be useful in this setting. The DWI distinguishes fibrosis from tumor invasion. Besides these limitations, VI-RADS does not provide validation for patient risk stratification, therapy selection, and the monitoring of therapeutic response [20].

Segmentation of bladder cancer on mp-MRI is the first step to non-invasively identifying bladder tumors and then evaluating muscle invasiveness and tumor stage. DL algorithms utilizing CNN have achieved remarkable success in the image segmentation field [21]. Three-dimensional deep CNN models have the potential to automate gross tumor volume (GTV) contouring on mp-MRI. However, there are some challenges with tumor contouring. The accuracy could depend on the radiologist’s experience, tumor heterogeneity, and whether tumor-to-normal tissue interference is poor or not. 

Motivated by this need, in this work, we evaluate emerging segmentation algorithms and compare their efficiency to each other to advance the bladder cancer mp-MRI efficiency.

## 2. Materials and Methods

### 2.1. Patients

This study was approved by the Institutional Review Board. We queried the institution’s medical records to obtain all patients who underwent mp-MRI for the diagnosis of bladder cancer between October 2015 and February 2023. We identified 217 cases and enrolled 53 patients in the study. The inclusion criteria were patients with pathologically confirmed bladder masses and pelvic imaging with a 3T MRI scanner. Exclusion criteria were no detectable tumor, insufficient MR images, severe imaging artifacts, and artificial devices in the imaging field.

### 2.2. Magnetic Resonance Imaging

Patients underwent MRI at one of three clinical scanners (Vida, Trio, or Skyra; Siemens Erlangen, Germany). The pelvic mp-MRI protocol encompassed high-resolution multiplane T2-weighted imaging (T2WI) with fat suppression, axial diffusion-weighted imaging (DWI), and axial T1-weighted contrast-enhanced (T1WI) sequences before and after contrast injection. Table 1 shows the sequence parameters used in the MRI protocol. T2WI used turbo spin echo acquisition. The DWI sequence used echo planar imaging (EPI) acquisition with b values = 0 and 500 s/mm^2^. Apparent diffusion coefficient (ADC) maps were automatically generated by the scanner software using all b-values. The T1-weighted imaging utilized a volumetric interpolated breath-hold examination (VIBE) sequence. A gadolinium-based contrast agent (DOTAREM, Bayer Pharma, and Berlin, Germany) was injected at a dose of 0.1mm/kg. Contrast-enhanced images were acquired in the following phases: arterial, venous, and delayed (3 min). 

### 2.3. Image Segmentation

**Figure 2:**
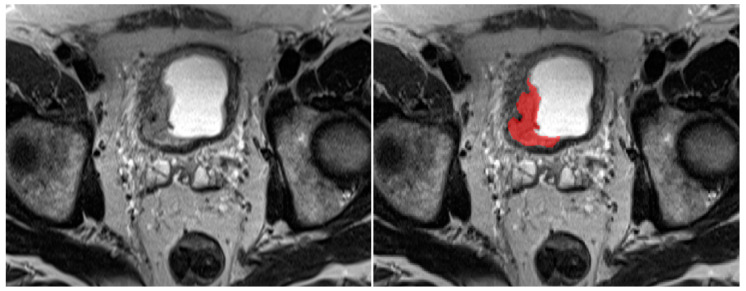
Axial T2-weighted image of a patient with bladder cancer and manual contouring of the tumor (red) on the right.

A fellowship-trained abdominal radiologist and MRI scientist with more than 10 years of experience manually segmented bladder tumors on each slice of the T2WI, ADC, and arterial phase T1WI images using ITK-SNAP 3.8.0 software (www.itksnap.org, USA, accessed on 1 February 2024) (Figure 2) [22]. Three masks were created for each patient. The segmentations were made in consensus by the investigators and considered as the ground truth, which means the AI models aimed to predict the segmentations during training and validation.

### 2.4. Deep Learning Models: Training and Evaluation

**Figure 3:**
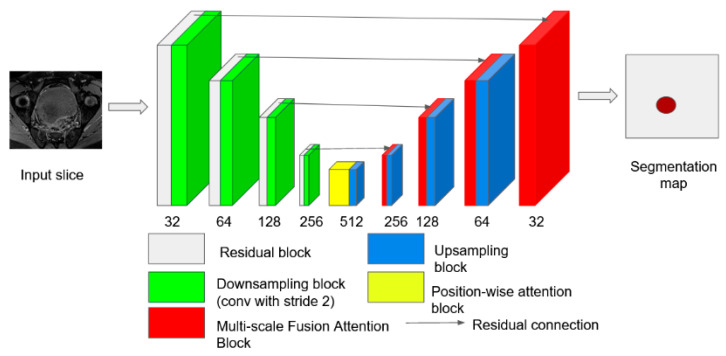
The architecture of the evaluated MAnet, a Unet variant, is shown for illustration. The multiscale fusion attention block merges the features from both encoding and decoding paths at multiple levels. Unlike Unet, which simply aggregates the features, the attention blocks learn how to merge more important regions, guided by the attention mechanisms.

We trained three existing models (MAnet, PSPnet, and Unet) to perform bladder tumor segmentation on mp-MRI images [23]. Unet is considered the backbone of medical image segmentation [24]. PSPnet and MAnet have gained popularity for image segmentation for scene understanding and improved contextual features, respectively [25]. These deep networks have encoding and decoding components. The encoding contracting component learns the visual localizing features, and then the decoding expanding pathway adaptively integrates local features with their global dependencies [24] (Figure 3).

Models were given a single MRI slice as input and trained to segment tumors based on manually drawn masks. Each model was trained on T2WI, ADC, and T1WI images separately. The epoch was set to 70.

Loss functions are a measure of how well an AI model’s prediction matches the true value. It quantifies the difference between the predicted value and the actual value. We used three popular loss functions during training: cross-entropy (CE), dice loss (DSC), and focal loss (FL) [26,27,28]. In the context of this work, let us introduce the following notation. Let $X\in R^{W\times H\times D}$ be a 3D input image, whose spatial dimension is width (W), height (H) and depth (D), and $Y\in {\left\{0,1\right\}}^{W\times H\times D\times K}$ its corresponding ground truth mask, with K being the total number of classes. The segmentation network is a function parameterized by θ, whose last layer is a SoftMax function producing the segmentation results $\hat{Y\in {\left\{0,1\right\}}^{W\times H\times D\times K}}$. Thus, the cross-entropy loss, which measures the similarity between the ground truth and probabilistic prediction distributions, for a given image, can be defined as follows:

$$L_{C}=-\frac{1}{D\times H\times W}\sum_{d=1}^{D}\sum_{i=1}^{H}\sum_{j=1}^{W}\sum_{k=1}^{K}\overset{\left(k\right)}{\underset{dij}{y}}\log\hat{y_{dij}^{\left(k\right)}}$$

The dice similarity coefficient (DSC) compares volumes based on their overlaps between the ground truth and the predicted image. During training, dice loss is leveraged to achieve a perfect match of DSC 1.0 [29], which resorts to minimizing the following loss:

$$L_{\mathrm{Dc}}=1-\frac{2\sum_{d=1}^{D}\sum_{i=1}^{H}\sum_{j=1}^{W}\sum_{k=1}^{K}y_{dij}^{\left(k\right)}\hat{y_{dij}^{\left(k\right)}}}{\sum_{d=1}^{D}\sum_{i=1}^{H}\sum_{j=1}^{W}\sum_{k=1}^{K}y_{dij}^{\left(k\right)}+\sum_{d=1}^{D}\sum_{i=1}^{H}\sum_{j=1}^{W}\sum_{k=1}^{K}\hat{y_{dij}^{\left(k\right)}}}$$

Last, focal loss aims to address the class imbalance on the image by increasing the focus of the model on the selected class, which is a tumor in this case [27]. This loss is formally defined as follows:

$$LF=-\frac{1}{D\times H\times W}\sum_{d=1}^{D}\sum_{i=1}^{H}\sum_{j=1}^{W}\sum_{k=1}^{K}{\left(1-\hat{y_{dij}^{\left(k\right)}}\right)}^{\gamma }y_{dij}^{\left(k\right)}\log\hat{y_{dij}^{\left(k\right)}}$$

where γ controls the rate at which easy samples (i.e., voxels) are down-weighted.

To evaluate the segmentation performance, we resorted to two popular metrics in the medical image segmentation literature, the dice similarities coefficient (DSC) and Hausdorff distance (HD), whereas we used the expected calibration error (ECE) to measure the calibration performance of the different models [30,31]. 

Due to the limited dataset size, we used a 4-fold validation strategy to validate the models’ performance. For each fold, we trained the models with 40 patients and tested them on the remaining 13 patients. The final validation metrics are the average of those for each fold.

## 3. Results

The patient ages ranged from 36 to 97 with a mean of 66.7 years. A total of 24 patients out of 53 had MIBC. Table 2 presents DSC, HD, and ECE values on T1WI, T2WI, and ADC testing datasets, obtained by evaluating different deep learning models, MAnet, PSPnet, and Unet, with different learning objectives: cross-entropy (CE), cross-entropy plus dice loss (CE+DSC), and focal Loss. These results show that, in terms of DSC values, MAnet with CE+DSC provided the highest DSC on T1WI, T2WI, and ADC images.

**Figure 4:**
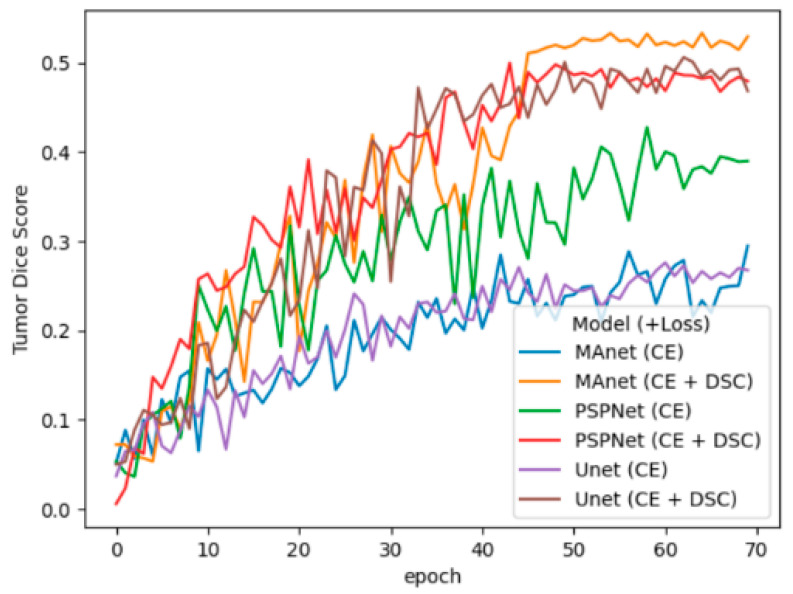
Dice score evolutions for tumor segmentation on the T1-weighted images. In the plots, we can observe that variants integrating the compound CE+DSC loss not only reach higher dice scores but also converge faster than the models using only CE as a loss function.

**Figure 5:**
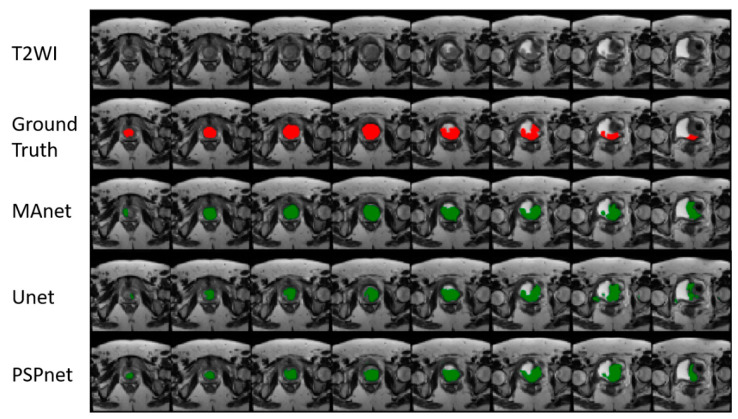
Visual results for tumor segmentation (green) achieved by the MAnet, Unet, and PSPnet models (loss function: CE+DSC) are compared to the manual segmentations in red (ground truth).

Figure 4 illustrates the evolution of DSC and the accuracy of both Unet (CE + DSC) and MAnet (CE+DSC) on a training validation set for tumor segmentation on T1WI. Figure 5 illustrates the performance of the models on a case in comparison to the manual segmentations (ground truth).

In terms of distance similarity, i.e., HD, PSPnet with CE+DSC obtained the smallest HD distances on the tumor segmentation across all modalities.

The expected calibration errors were the smallest for PSPnet with FL on the ADC images, whereas they were the smallest for MAnet with CE+DSC on T2WI and T1WI, which indicates that this model yields the most reliable predictions among the analyzed ones.

Overall, the models achieved better segmentation on the ADC and T1WI than on the T2WI. 

## 4. Discussion

In this study, we trained the Unet, PSPnet, and MAnet networks with three loss functions to segment bladder cancer on the mp-MRI images and assessed their performance [24]. MAnet with CE+DSC gave the best DSC values on all images. On the other hand, PSPnet with CE+DSC achieved the smallest HD distances on all images. These results indicate that compared to Unet, MAnet and PSPnet showed better performances in BC segmentation depending on the choice of the loss function and evaluation metrics.

Loss functions are used to quantify the error between the predicted and actual data. For bladder segmentation, we studied the three most commonly used loss functions: CE, CE+DSC, and FL. Among them, we observed that CE+DSC provided the best training when DSC was used as an evaluation metric. These experiments show that hybrid loss functions such as CE+DSC can be more effective than single ones. This can be attributed to better handling of the class imbalance problem [30,32]. Class imbalance refers to an unequal distribution of foreground and background elements in the image. This is a big issue in bladder MRI where a tumor is too small compared to the rest of the image [27]. Overall, these results highlight the careful selection of loss functions in training segmentation algorithms.

When it comes to choosing evaluation metrics, compared to ECE, both DSC and HD are more commonly used in performance evaluations of DL models in medical image segmentation. DSC is a popular metric that assesses the similarity (overlapping) between the model-predicted area and the reference area [26]. On the other hand, HD is a distance-based metric that shows how far two contours are from each other [30]. We observed that DSC and HD favored different models in BC segmentation. While MAnet gave better DSC scores, PSPnet gave lower HD values. We also evaluated the confidence of the models using ECE. ECE indicates how reliable a model is by comparing the model predictions with the true outputs [31]. A low ECE value denotes a better-calibrated model. Our experiments demonstrated that PSPnet with FL had the lowest ECE on the ADC images, whereas MAnet with CE+DSC had the smallest ECEs on the T1WI and T2WI. This could indicate that combined metrics such as CE+DSC in the training step could reduce calibration errors as well.

Notably, the DL models consistently achieved better tumor segmentation on the ADC and T1WI than on the T2WI images, which could be explained by contrast differences among these sequences. The gadolinium-based contrast agent enhances tumor signal on T1-weighted images, while DWI results in hypointensity on the ADC images in case of diffusion restriction. These contrast mechanisms allow a clear distinction of tumors on these sequences. On the other hand, the contrast on T2WI relies on T2 relaxation of tissues, which results in a low tumor signal compared to background tissue. The contrast to noise ratio (CNR) based on T2 relaxation might not be as distinctive as the CNRs on the T1WI and ADC mechanisms. 

In the literature, Dolz et al. (2018) conducted one of the earlier works on the segmentation of BC on MRI. They demonstrated the feasibility of fully automated segmentation of the bladder mass, inner wall, and outer wall on T2-weighted images. [33]. Dolz et al. introduced progressive dilated convolutions in each convolutional block to increase the receptive fields for the first time. Later, Yu et al. proposed the Cascade Path Augmentation Unet (CPA-Unet) network, which mines multiscale features [34]. They showed effective segmentation of the bladder tumor, inner wall, and outer wall on T2-weighted images. Recently, Moribate et al. used a modified Unet model to segment tumors on DWI (b0 and b1000) and ADC images [35]. They tested the b0, b1000, ADC, and multi-sequence (b0-b1000-ADC) images as input in their model. They reported the highest dice similarity coefficient for multi-sequence images as input. Compared to these studies, our work presents new findings in the following aspects: First, while previous work focused on the segmentation of tumors on a single MRI sequence such T2WI or ADC, we report the segmentation results for three sequences in the mp-MRI protocol: T2WI, T1WI, and ADC. Second, rather than a single DL model, we present comparative performances of three prominent DL models in medical image segmentation. Lastly, we report the segmentation accuracies based on three loss functions and two evaluation metrics along with calibration errors.

Although the models compared in this study yielded promising results in segmenting bladder tumors, the overall accuracy was less than previously reported MRI studies and the accuracies in other modalities such as computed tomography (CT). We believe this arises from mainly the small sample size. Fifty-three patients are relatively small in DL training; hence, our results can be considered preliminary. Moreover, the segmentation of the bladder mass on mp-MRI is challenging. The human bladder is a hollow distensible organ, presenting a variety of volumes, shapes, and positions. This is a challenge, as the model needs to learn a large variety of features to train properly. Tumor variability with its various shapes and sizes produces another set of variabilities. To capture all of these variabilities, many clinical cases are needed. Also, it is common to have various artifacts on mp-MRI images due to urine flow, magnetic field inhomogeneities, etc., which makes segmentation difficult compared to CT [36,37,38,39]. 

This preliminary study has several limitations. First, the sample size was small and only from a single institution, limiting the models from learning the high variability of bladder tumors. Second, the MRI datasets were only from 3T scanners, limiting the generalizability of the results to other magnetic field strengths. Third, we tested the models separately on each sequence. Future work will encompass inputting the images from three sequences at the same time to the models. We will also study predicting the muscle invasiveness of tumors based on segmented MRI images in future studies.

## 5. Conclusions

In conclusion, MAnet and PSPnet models could show promising success in the automatic segmentation of bladder tumors, which constitutes the first step toward determining muscle invasiveness. However, the accuracy of the models also relies on the careful selection of loss functions during training and the right choice of evaluation metrics. Among the three MRI sequences, the segmentation accuracy was better on the ADC and T1WI compared to T2WI. To evaluate the true potential of these networks in segmenting BC on mp-MRI images, larger datasets from multiple institutions are needed.

## Figures and Tables

**Table 1:** MRI parameters for the used sequences.

	T2WI (Axial)	T1WI DCE (Axial)	DWI (Axial)
Repetition Time (TR), ms	5970	2.96	TR = 4600
Echo Time (TE), ms	86	1.18	TE = 84
Field of View (FOV), mm	199 × 199	240 × 240	220 × 260
Matrix	448 × 448	256 × 256	136 × 160
Slice Thickness (ST), mm	3	3	4
b-value, s/mm^2^	-	-	0 and 500

**Table 2:** Tumor segmentation performance of the three models (MAnet, PSPnet, and Unet), which were trained using three loss functions (CE, CE+DSC, and FL), were evaluated based on DSC, HD, and ECE on the T2WI, ADC, and T1WI images.

						Tumor				
			DSC			HD			ECE	
Model	Modality Loss	ADC	T1	T2	ADC	T1	T2	ADC	T1	T2
MAnet	L*CE*	0.4550	0.3175	0.2875	47.54	67.62	76.91	0.0200	0.0200	0.0350
	L*CE* + L*DSC*	0.5925	0.5600	0.4650	23.92	27.52	41.23	0.0150	0.0075	0.0100
	L*FL*	0.3700	0.2575	0.2450	46.83	72.58	74.64	0.0525	0.1025	0.1125
PSPnet	L*CE*	0.4200	0.4500	0.2700	52.84	56.28	78.27	0.0175	0.0250	0.0325
	L*CE* + L*DSC*	0.5650	0.5075	0.4175	10.71	21.94	31.34	0.0150	0.0175	0.0200
	L*FL*	0.3825	0.3925	0.2600	47.26	45.68	80.85	0.0125	0.0150	0.0250
Unet	L*CE*	0.4950	0.2900	0.2750	39.21	87.53	85.57	0.0200	0.0200	0.0250
	L*CE* + L*DSC*	0.5825	0.5250	0.4525	33.86	46.09	57.34	0.0150	0.0075	0.0150
	L*FL*	0.3850	0.2725	0.2100	36.32	85.04	82.97	0.0475	0.0925	0.1275

## Data Availability

The datasets presented in this article are not publicly available due to restrictions to protect the privacy of study participants.
